# Rapid and sensitive detection of human astrovirus in water samples by loop-mediated isothermal amplification with hydroxynaphthol blue dye

**DOI:** 10.1186/1471-2180-14-38

**Published:** 2014-02-14

**Authors:** Bo-Yun Yang, Xiao-Lu Liu, Yu-Mei Wei, Jing-Qi Wang, Xiao-Qing He, Yi Jin, Zi-Jian Wang

**Affiliations:** 1 School of Biological Sciences and Technology, Beijing Forestry University, Qinghua East Rd 35, P. O. Box 162, Haidian District, Beijing 100083, P. R. China; 2 State Key Laboratory of Environmental Aquatic Chemistry, Research Center for Eco-Environmental Sciences, Chinese Academy of Sciences, P. O. Box 2871, Beijing 100085, P. R. China

**Keywords:** Astrovirus, Loop-mediated isothermal amplification, Hydroxynaphthol blue

## Abstract

**Background:**

The aim of this paper was to develop a reverse transcription loop-mediated isothermal amplification (RT-LAMP) method for rapid, sensitive and inexpensive detection of astrovirus.

**Results:**

The detection limit of LAMP using *in vitro* RNA transcripts was 3.6×10 copies·μL^-1^, which is as sensitive as the presently used PCR assays. However, the LAMP products could be identified as different colors with the naked eye following staining with hydroxynaphthol blue dye (HNB). No cross-reactivity with other gastroenteric viruses (rotavirus and norovirus) was observed, indicating the relatively high specificity of LAMP. The RT-LAMP method with HNB was used to effectively detect astrovirus in reclaimed water samples.

**Conclusions:**

The LAMP technique described in this study is a cheap, sensitive, specific and rapid method for the detection of astrovirus. The RT-LAMP method can be simply applied for the specific detection of astrovirus and has the potential to be utilized in the field as a screening test.

## Background

Human astroviruses (HAstV) have been shown in several epidemiologic outpatient studies to be an important cause of viral gastroenteritis in infants and young children. HAstV have been associated with outbreaks in day-care centers for children and adults [1]. The incidence of astrovirus infections has been estimated at between 5% and 10% in children with gastroenteritis [2]. The reported frequency of infection by astrovirus was 8% during the winter season (from December 2000 to March 2001) in Beijing [3].

Astroviruses are among the most resistant viruses; they show resistance against different physical and chemical agents, they are able to maintain their infectivity at 60°C for 10 min, and they are resistant to treatment at pH 3 [4]. Astroviruses spread via the fecal–oral route, through direct personal contact, or via contaminated food and water, and they have been reported to affect otherwise healthy people exposed to astrovirus-contaminated food or water [1]. However, the number of reports on astrovirus detection is relatively low.

Several detection methods have been developed to detect the presence of astrovirus in clinical isolates, raw sewage samples, groundwater and surface water, including cell culture [1], enzyme immunoassay and nucleotide sequencing [5], and PCR-based assays [4]. All of these methods are effective and accurate in detecting the virus infection in the laboratory. However, these methods have some intrinsic disadvantages such as the requirement for expensive equipment and reagents, and being laborious and time consuming, and are thus unfavorable for use on a wide scale. A detection method that is not only rapid and sensitive, but also simple and economical to handle, is needed for practical application.

To meet these requirements, a reverse transcription loop-mediated isothermal amplification (RT-LAMP) reaction was developed as an alternative method. The LAMP assay is a rapid, accurate and cost-effective diagnostic method that amplifies the target nucleic acid under isothermal conditions, usually between 60°C and 65°C [6]. Hence, only simple equipment such as a heating block or a water bath is required. The final products of the RT-LAMP reaction are DNA molecules with a cauliflower-like structure and multiple loops consisting of several repeats of the target sequence [7]. LAMP has been applied for the specific detection of aquatic animal viruses such as foot-and-mouth disease virus [8], Singapore grouper iridovirus [9] and H1N1 2009 virus [10,11].

The LAMP reaction results in large amounts of pyrophosphate ion byproduct. These ions react with Mg^2+^ ions to form the insoluble product, magnesium pyrophosphate. Because the Mg^2+^ ion concentration decreases as the LAMP reaction progresses, the LAMP reaction can be quantified by measuring the Mg^2+^ ion concentration in the reaction solution [12]. Hydroxynaphthol blue (HNB) is used for colorimetric analysis of the LAMP reaction. The HNB dye-based assay has a remarkable advantage compared with other color-based assays [11,12] in that HNB is mixed prior to amplification. The need to open the assay samples to add the dye is thereby omitted, thus reducing the risk of cross-contamination.

HAstV is classified into eight distinct antigenic serotypes, HAstV 1–8, with serotype 1 predominating in most countries [13]. HAstV-1 was also identified as the predominant serotype in China [14]. Wei et al. [13] developed a one-step, real-time reverse-transcription LAMP (rRT-LAMP) method with a turbidimeter targeting the 5’ end of the capsid gene for rapid and quantitative detection of HAstV-1 from stool specimens. In our study, RT-LAMP with HNB for specific, rapid and sensitive detection of HAstV-1 in water samples was developed. To our knowledge, this is the first report of the application of RT-LAMP with HNB to HAstV-1.

## Results

### Optimized LAMP reaction

The LAMP reaction was performed using plasmid DNA as template to determine the optimal reaction conditions. The optimal concentrations of betaine and Mg^2+^ ion in the LAMP reactions were 1 mmol·L^-1^ and 4 mmol·L^-1^, respectively (data not shown). The amplicon was formed at 63, 64, 65 and 66°C, with the optimal temperature for product detection being 65°C. Thus, 65°C was used as the optimum temperature for the following assays. Although we could detect well-formed bands at 60 min, the reaction time was extended to 90 min to ensure positive detection of lower concentration templates in the system.

### Naked-eye observation of LAMP products using HNB

**Figure 1:**
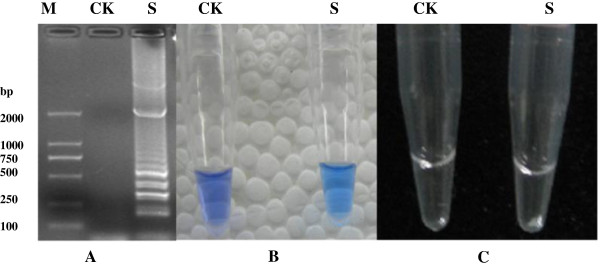
**Detection of LAMP products by observation of white turbidity and the color of the reaction mixture. (A)** LAMP detection of astrovirus by electrophoresis; **(B)** Color reaction with HNB; **(C)** White precipitates M: Marker; CK: Blank control; S: Astrovirus.

The LAMP reaction was incubated in a conventional water bath at 65°C for 90 min, followed by heating at 80°C for 2 min to terminate the reaction. The ability to detect astrovirus LAMP products using HNB was examined. Positive amplification was indicated by a color change from violet to sky blue, as shown in Figure 1B, and verified by agarose gel electrophoresis (Figure 1A) and white precipitates (Figure 1C). The positive color (sky blue) was only observed with the reference virus, whereas none of the control viruses showed a color change.

### Specificity and sensitivity of the LAMP assay

**Figure 2:**
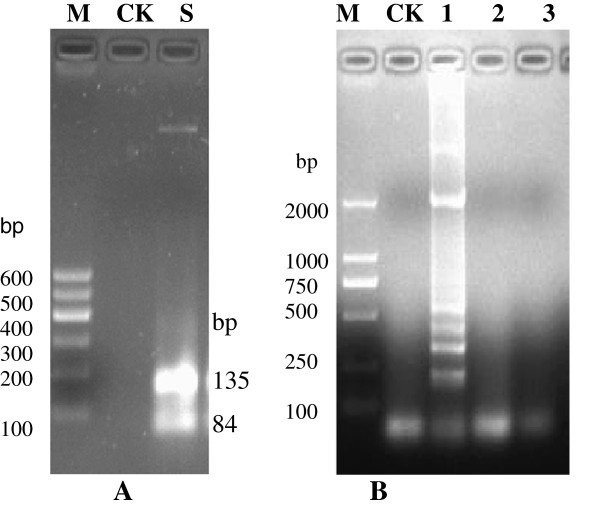
**Specificity of astrovirus detection using the LAMP assay. (A)** Restriction analysis; **(B)** Specificity analysis of cross-reaction by electrophoresis M: Marker; CK: Blank control; S: LAMP products after digestion with EcoNI 1: Astrovirus; 2: Rotavirus; 3: Norovirus.

The sizes of the LAMP fragments digested with the restriction enzyme, EcoN1, were analyzed by electrophoresis, and the results showed agreement with the predicted sizes of 84 and 135 bp (Figure 2A). The specificity of the LAMP assays was examined with two other enteric viruses: rotavirus and norovirus. The results of the LAMP assay were positive for astrovirus and negative for rotavirus and norovirus (Figure 2B).

**Figure 3:**
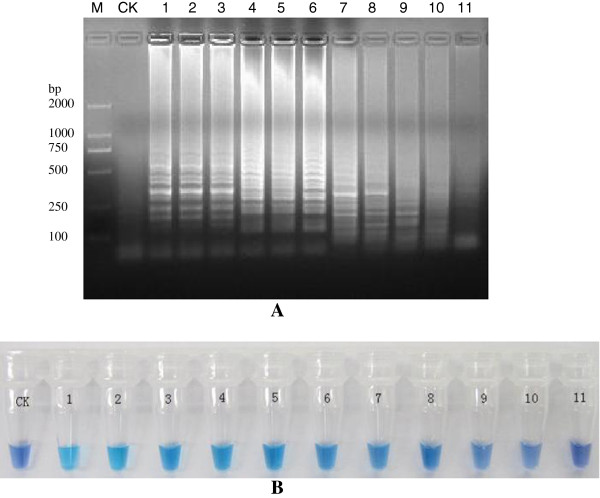
**Sensitivity analysis of LAMP detection of astrovirus. (A)** Electrophoresis; **(B)** Color reaction with HNB M: marker; CK: Blank control; 1: Astrovirus RNA 3.6 × 10^9^ copies·μL^-1^; 2: 3.6 × 10^8^ copies·μL^-1^; 3: 3.6 × 10^7^ copies·μL^-1^; 4: 3.6 × 10^6^ copies·μL^-1^; 5: 3.6 × 10^5^ copies·μL^-1^; 6: 3.6 × 10^4^ copies·μL^-1^; 7: 3.6 × 10^3^ copies·μL^-1^; 8: 3.6 × 10^2^ copies·μL^-1^; 9: 3.6 × 10 copies·μL^-1^; 10: 3.6 copies·μL^-1^; 11: 3.6 × 10^-1^ copies·μL^-1^.

The reaction was tested using 5 μL of 10-fold serial dilutions of *in vitro* RNA transcripts (3.6×10^9^ copies·μL^-1^) and compared with PCR assays. The detection limit of LAMP using astrovirus RNA was 3.6× 10 copies·μL^-1^ (Figure 3A and B), and the LAMP reaction followed by colorimetric analysis could be completed within 100 minutes. Similarly, the detection limit of PCR was also 3.6× 10 copies·μL^-1^, but followed by gel electrophoresis required about 3 h for completion (data not shown).

### Evaluation of RT-LAMP assay with reclaimed water samples

**Figure 4:**
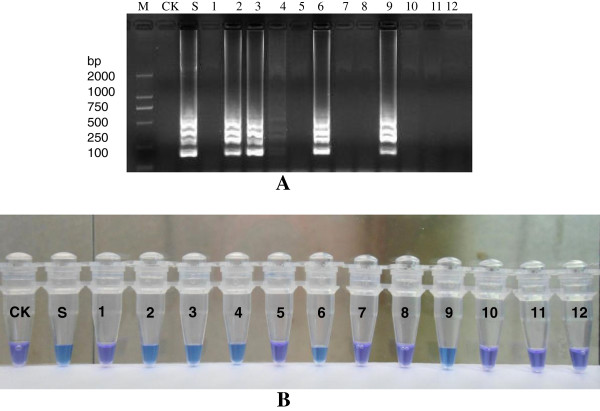
**LAMP for detection of astrovirus in water samples. (A)** Electrophoresis **(B)** Color reaction with HNB M: Marker; CK: Blank control; S: Astrovirus; 1-12: Samples.

Comparative evaluation of RT-LAMP with routine RT-PCR was performed to examine astrovirus in 12 reclaimed water samples. Five samples (No. 2, 3, 4, 6, 9) were positive and the frequency of astrovirus detection was 41.7% (5/12) with RT-LAMP (Figure 4A and B). In contrast, four samples (No. 2, 3, 6, and 9) were positive and the frequency of astrovirus detection was 33.3% (4/12) with RT-PCR (data not shown). This may indicate that the astrovirus RT-LAMP assay is slightly more sensitive than RT-PCR for the detection of astrovirus in water samples with very low viral titers.

## Discussion

This study demonstrated that the LAMP method described here for astrovirus detection is highly sensitive, and can detect as few as 3.6× 10 copies·μL^-1^ of astrovirus template RNA. The detection limit of the RT-LAMP assay with HNB for pandemic influenza A H1N1 virus was approximately 60 copies in a 25 μL reaction mixture [11]. Detection of target DNA by LAMP compared with detection by PCR was at least equivalent or more sensitive [9]. This was confirmed by results showing that the detection limit of LAMP was as sensitive as the currently used PCR assays for the detection of astrovirus.

Though DNA plasmid is served as template for optimizing virus detection assays in some cases [13] since RNA molecules are not stable *in vitro*. However, plasmid DNA is not fully representative of RNA viruses such as astrovirus. And RNA transcripts *in vitro* will be better served as a template for the optimization of this assay. We completed the sensitivity analysis using *in vitro* RNA transcripts, which will provide important comparative reference to other laboratories doing similar research.

In this study, we only compared the specificity of the reaction for astrovirus, rotavirus and norovirus because the reported frequencies of infection by rotavirus, astrovirus and norovirus are 59%, 8% and 6%, respectively, in Beijing [3]. Astrovirus, rotavirus and norovirus are the top three viruses associated with diarrhea. Regarding the specificity among different serotypes of astrovirus, a similar study using primers from the same conserved capsid protein (ORF2) gene of HAstV-1 indicated that the LAMP assay had a high degree of specificity for HAstV-1 [13]. In future experiments, we will synthesize the target sequences of HAstV 2-8 and transcribe them *in vitro*. The resulting RNA segments will then be used to investigate cross-reactivity with the HAstV-1-specific LAMP primers.

The use of HNB for visual inspection of LAMP amplification products was a simple and effective technique, with no gel electrophoresis and staining with ethidium bromide required. Hence, LAMP is a superior method in terms of its economic feasibility and safety. The HNB dye-based assay has a remarkable advantage compared with other color-based assays because (i) opening the reaction tube is not required to determine whether the reaction is positive or negative (this reduces the risk of cross-contamination); (ii) the detection sensitivity is equivalent to that of SYBR green assays; and (iii) the positive/negative result of the LAMP reaction can be easily judged with the naked eye [12]. This colorimetric assay is superior to the existing colorimetric assays for LAMP with regard to reducing contamination risks, and is helpful in high-throughput DNA and RNA detection [12]. Thus, RT-LAMP with HNB dye was shown to be a sensitive and simple assay for detection of many viruses [11]. Although quantitative detection is difficult, inspection with the naked eye was simple and rapid. Therefore, it may facilitate the application of LAMP as a field test [9].

Using the LAMP assay, we were able to detect astrovirus in various environmental water samples with a simple water bath. A water bath is the only equipment needed, and is used for both the DNA preparation and nucleic acid amplification. With no complicated equipment and technical training, LAMP is very simple to perform and offers advantages compared with other techniques [9]. Additional studies, including improvements in sensitivity and validation of visual testing with a larger number of water samples, are necessary before this method can be applied widely for routine testing both in the laboratory and in the field. The simplicity, ease of use and cost-effectiveness of this method makes it an attractive assay for the rapid screening of human astrovirus.

## Conclusions

The LAMP technique described in this study is a cheap, sensitive, specific and rapid method for the detection of astrovirus. The RT-LAMP method can be simply applied for the specific detection of astrovirus and has the potential to be utilized in the field as a screening test.

## Methods

### Design of RT-LAMP primers

**Figure 5:**
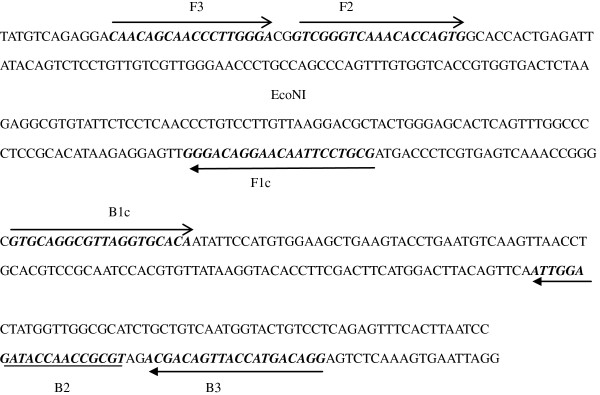
Oligonucleotide primers used for RT-LAMP of astrovirus.

A set of four species-specific RT-LAMP primers was designed to target the HAstV-1 capsid protein gene (ORF2), as described by Guo et al. [5,14]. The RT-LAMP primers were designed using the Primer Explorer 4.0 software program (http://primerexplorer.jp/e/), and were named as follows: forward outer primer, F3; backward outer, B3; forward inner primer, FIP; and backward inner primer, BIP. The primer sequences and their locations are indicated in Table 1 and Figure 5.

**Table 1:** Sequences of the primers used

**Primer name**	**Type**	**Sequence (5' → 3')**
F3	Forward outer	CAACAGCAACCCTTGGGA
B3	Backward outer	GGACAGTACCATTGACAGCA
FIP	Forward inner prime (F1_C_-TTTT-F2)	GCGTCCTTAACAAGGACAGGGTTTTTGTCGGGTCAAACACCAGTG
BIP	Backward inner primer (B1_C_-TTTT-B2)	GTGCAGGCGTTAGGTGCACATTTTTGCGCCAACCATAGAGGTTA

### Construction of the pGH plasmid

A recombinant plasmid, pGH-A-X3178G, was constructed as a template for the development of the astrovirus RT-LAMP protocol. A 449 bp astrovirus ORF2 DNA segment (GenBank accession number, GQ169035.1) was amplified and cloned into the pGH vector (AOKE Bio Co. Ltd., Beijing, China) according to the manufacturer’s instructions. The DNA segment spanned the sequences between the F3 and B3 primers.

### LAMP reaction

The preliminary LAMP for the astrovirus DNA in the plasmid template was carried out in a 25 μl reaction containing 0.2 μmol·L^-1^ each of F3 and B3, 1.6 μmol·L^-1^ each of FIP and BIP, l mmol·L^-1^ dNTPs, l mol·L^-1^ betaine, 6 mmol·L^-1^ MgSO_4_, 2.5 μL 10× Bst-DNA Polymerase Buffer, 8 U Bst DNA polymerase (NEB, Beijing, China) and 5 μL template DNA. The reaction time was optimized by incubating the mixture for 15, 30, 60, 90 and 120 min at 65°C, while the reaction temperature was optimized by incubating the mixture at 60, 61, 62, 63, 64, 65 and 66°C for 60 min. The concentrations of betaine and Mg^2+^ ion in the LAMP reaction solutions were optimized using a series of concentrations from 1 mol·L^-1^ to 4 mol·L^-1^ and from 1 mmol·L^-1^ to 7 mmol·L^-1^, respectively. The reaction was terminated by heating at 80°C for 5 min. The LAMP products (5 μL) were electrophoresed on 1.5% agarose gels and stained with GoldView to determine the optimal conditions. The possibility of LAMP detection of astrovirus using HNB (Lemongreen, Shanghai, China) was examined. HNB was dissolved in distilled water at 1.5 mM to prepare a stock solution, and the reaction solution contained a final HNB concentration of 120 μM [12]. For the sensitivity assay and reclaimed water, 1 μL avian myeloblastosis virus reverse-transcriptase (10 U/μL, Invitrogen, Carlsbad, USA) was added to the reaction mixture.

### PCR detection

PCR amplification of astrovirus DNA in plasmids was performed using the following reaction conditions: 5 μL 10× Ex-Taq buffer, 50 μmol·L^-1^ dNTPs, 0.12 μmol·L^-1^ F3, 0.12 μmo ·L^-1^ B3, 2.5 U Ex-Taq DNA polymerase (TaKaRa, Dalian, Chian), 10 μL template DNA. Amplification conditions were as follows: 94°C, 3 min; 40 cycles of 30 s at 94°C, 30 s at 50°C and 1 min at 72°C; with a final extension of 5 min at 72°C. A positive control and a negative control (nuclease-free water) were included for each PCR assay. The amplified DNA fragments were analyzed by electrophoresis on a 1.5% agarose gel and stained with GoldView. For the sensitivity assay and reclaimed water, 1 μL avian myeloblastosis virus reverse-transcriptase (10 U/μL, Invitrogen, Carlsbad, USA) was added into the reaction mixture.

### Specificity of the LAMP assay

The specificity of the assay was tested using DNA from astrovirus and two other enteric viruses as templates, including rotavirus and norovirus. In order to confirm the specificity of the LAMP reaction, the LAMP products were digested with the restriction enzyme, EcoN1 (NEB, Beijing, China), electrophoresed on 1.5% agarose gels and stained with GoldView. Based on theoretical calculations, the sizes of the main bands cut by EcoN1 should be 84 bp and 135 bp.

### Sensitivity of the LAMP assay

The detection limits of the rotavirus LAMP assay were evaluated using 10-fold serial dilutions of *in vitro* RNA transcripts. The astrovirus RNA (3.6×10^9^ copies·μL^-1^) was 10-fold serially diluted and 5 μL of each dilution was used as a template for the LAMP reaction. The optimum concentrations of betaine and Mg^2+^ ion determined as described above were added to the reaction mix. The reaction was performed at 65°C for 90 min and compared with a PCR assay.

### Application of RT-LAMP for the detection of astrovirus in reclaimed water samples

Twelve reclaimed water samples previously collected from sewage treatment plants were selected for RT-LAMP analysis. Two-liter samples of surface water were collected in sterile bottles and transferred to the laboratory, where they were immediately stored at 4°C for viral and bacterial investigations. A modified method developed for concentrating viruses in effluent from sewage treatment plants, including reclaimed water, was used to concentrate the water samples [15]. RNA was extracted using the Qiagen Viral RNA Extraction Kit (Qiagen, Germany) according to the manufacturer’s instructions, as described previously [16]. The 50 μl RNA eluates were stored at -80°C prior to amplification of nucleic acid. RT-PCR was carried out as control assay.

## Competing interests

All the authors declared that they have no competing interests.

## Authors’ contributions

XQH had full access to all of the data in the study and take responsibility for the integrity of the data and the accuracy of the data analysis; BYY and XLL performed the collection of water samples and the experiments; XQH wrote the majority of the manuscript; YMW, JQW and YJ supplied technical or material support. ZJW helped to revise the manuscript. All authors read and approved the final manuscript.

## References

[B1] Espinosa AC Mazari-Hiriart M Espinosa R Maruri-Avidal L Mendez E Arias CF Infectivity and genome persistence of rotavirus and astrovirus in groundwater and surface water Water Res 2008 42 10–11 2618 2628 18291437 10.1016/j.watres.2008.01.018

[B2] Mendez E Arias CF Knipe DM, Howley PM, Griffin DE, Lamb RA, Martin MA, Roizman B, Straus SE Astroviruses Fields Virology 2007 5 Philadelphia PA: Lipincott Willimas and Wilkins 981 1000

[B3] Liu C Grillner L Jonsson K Linde A Shen K Lindell AT Wirgart BZ Johansen K Identification of viral agents associated with diarrhea in young children during a winter season in Beijing J Clin Virol 2006 35 69 72 10.1016/j.jcv.2005.04.007 15998600 PMC7185874

[B4] Meleg E Jakab F Kocsis B B¨¢nyai K Melegh B Szcs G Human astroviruses in raw sewage samples in Hungary J Appl Microbiol 2006 101 5 1123 1129 10.1111/j.1365-2672.2006.02997.x 17040236

[B5] Noel JS Lee TW Kurtz JB Glass RI Monroe SS Typing of human astroviruses from clinical isolates by enzyme immunoassay and nucleotide sequencing J Clin Microbiol 1995 33 4 797 801 7790440 10.1128/jcm.33.4.797-801.1995 PMC228043

[B6] Tomita N Mori Y Kanda H Notomi T Loop-mediated isothermal amplification (LAMP) of gene sequences and simple visual detection of products Nat Protoc 2008 3 5 877 882 10.1038/nprot.2008.57 18451795

[B7] Notomi T Okayama H Masubuchi H Yonekawa T Watanabe K Amino N Hase T Loop-mediated isothermal amplification of DNA Nucleic Acids Res 2000 28 12 e63 10.1093/nar/28.12.e63 10871386 PMC102748

[B8] Dukes J King D Alexandersen S Novel reverse transcription loop-mediated isothermal amplification for rapid detection of foot-and-mouth disease virus Arch Virol 2006 151 6 1093 1106 10.1007/s00705-005-0708-5 16453084

[B9] Mao X Zhou S Xu D Gong J Cui H Qin Q Rapid and sensitive detection of Singapore grouper iridovirus by loop-mediated isothermal amplification J Appl Microbiol 2008 105 2 389 397 10.1111/j.1365-2672.2008.03761.x 18312563

[B10] Kubo T Agoh M Mai LQ Fukushima K Nishimura H Yamaguchi A Hirano M Yoshikawa A Hasebe F Kohno S Development of a reverse transcription-loop-mediated isothermal amplification assay for detection of pandemic (H1N1) 2009 virus as a novel molecular method for diagnosis of pandemic influenza in resource-limited settings J Clin Microbiol 2010 48 3 728 735 10.1128/JCM.01481-09 20071551 PMC2832456

[B11] Ma X Shu Y Nie K Qin M Wang D Gao R Wang M Wen L Han F Zhou S Visual detection of pandemic influenza A H1N1 Virus 2009 by reverse-transcription loop-mediated isothermal amplification with hydroxynaphthol blue dye J Virol Methods 2010 167 2 214 217 10.1016/j.jviromet.2010.03.027 20381535

[B12] Goto M Honda E Ogura A Nomoto A Hanaki KI Colorimetric detection of loop-mediated isothermal amplification reaction by using hydroxy naphthol blue Biotechniques 2009 46 3 167 172 10.2144/000113072 19317660

[B13] Wei H Zeng J Deng C Zheng C Zhang X Ma D Yi Y A novel method of real-time reverse-transcription loop-mediated isothermal amplification developed for rapid and quantitative detection of human astrovirus J Virol Methods 2013 188 1–2 126 131 23274752 10.1016/j.jviromet.2012.11.040

[B14] Guo L Xu X Song J Wang W Wang J Hung T Molecular characterization of astrovirus infection in children with diarrhea in Beijing, 2005–2007 J Med Virol 2010 82 3 415 423 10.1002/jmv.21729 20087940 PMC7166319

[B15] Katayama H Shimasaki A Ohgaki S Development of a virus concentration method and its application to detection of enterovirus and Norwalk virus from coastal seawater Appl Environ Microbiol 2002 68 3 1033 1039 10.1128/AEM.68.3.1033-1039.2002 11872447 PMC123733

[B16] He XQ Cheng L Li W Xie XM Ma M Wang ZJ Detection and distribution of rotavirus in municipal sewage treatment plants (STPs) and surface water in Beijing J Environ Sci Health A Tox Hazard Subst Environ Eng 2008 43 4 424 429 10.1080/10934520701795731 18273749

